# P-1361. High Dose Daptomycin Shows Non-Inferior Outcome Compared to Linezolid in Patients with Daptomycin and Vancomycin Resistant Enterocci Bloodstream Infection

**DOI:** 10.1093/ofid/ofaf695.1548

**Published:** 2026-01-11

**Authors:** Wei-Ting Lin, Yu-Chung Chuang, Jann-Tay Wang, Shan-Chwen Chang

**Affiliations:** National Taiwan University Hospital, Taipei City, Taipei, Taiwan (Republic of China); National Taiwan University Hospital, Taipei City, Taipei, Taiwan (Republic of China); Division of Internal Medicine, National Taiwan University Hospital, Taipei, Taiwan, Taipei, Taipei, Taiwan; Division of Internal Medicine, National Taiwan University Hospital, Taipei, Taiwan, Taipei, Taipei, Taiwan

## Abstract

**Background:**

Vancomycin-resistant Enterococcus (VRE), causes severe nosocomial infections and increase healthcare costs. Guidelines recommend high-dose daptomycin or linezolid for VRE bloodstream infections (BSI). Daptomycin-resistant VRE, emerging recently, limits treatment options. This study analyzes clinical characteristics, prognostic factors, and daptomycin and linezolid outcomes in daptomycin-resistant VRE BSI.

**Table 1.**
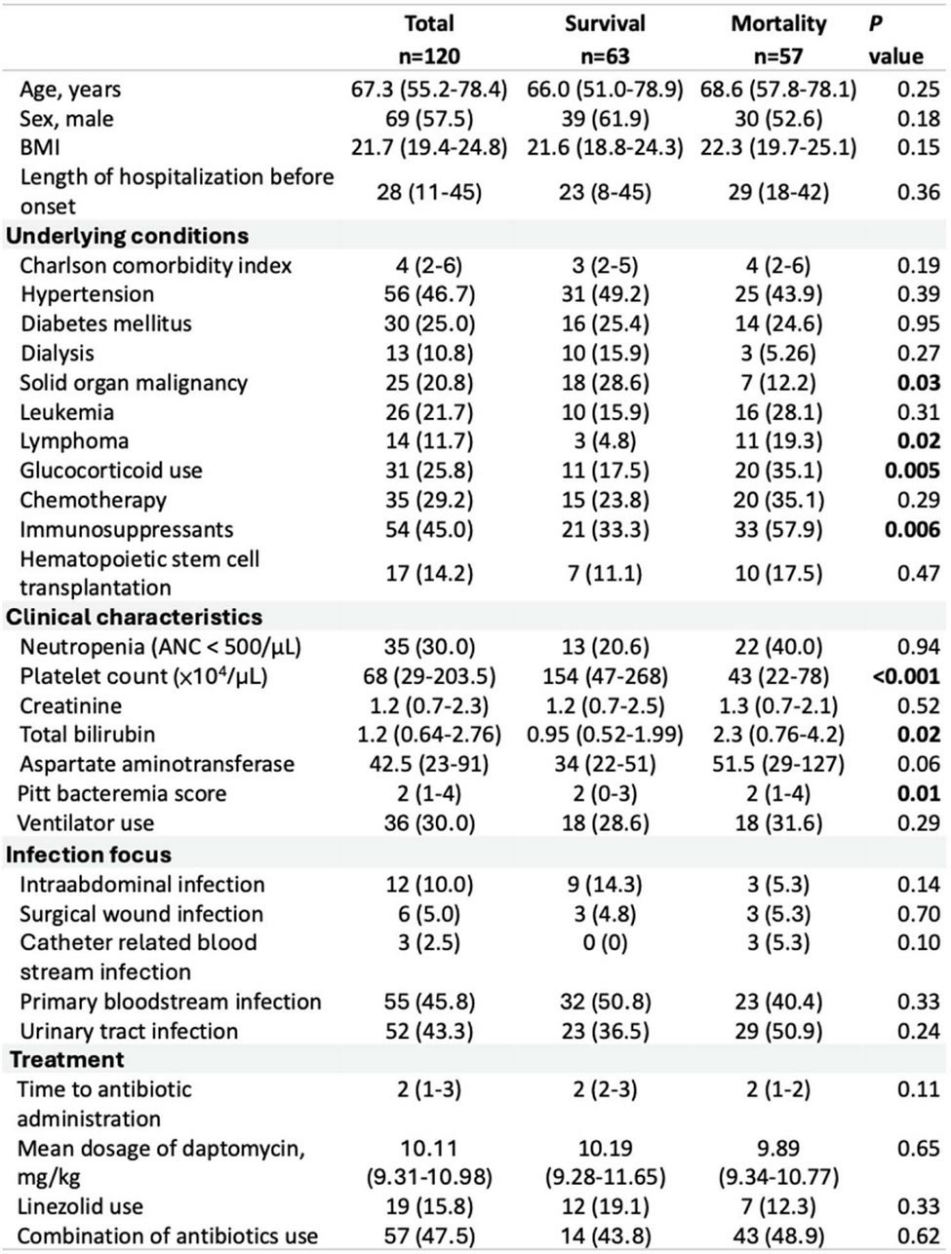
Baseline Characteristics of Included Subjects Note: Continuous variables are presented as median (IQR); categorical variables as number (%).

**Table 2.**
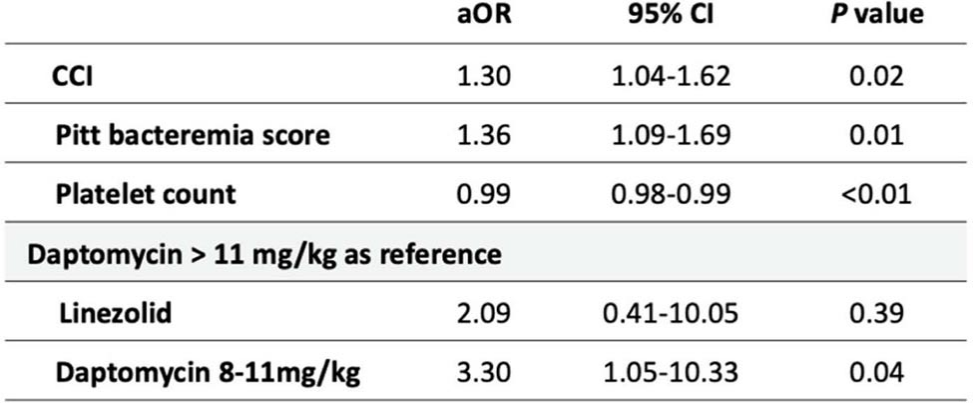
Multivariable Logistic Regression Analysis of 28-day Mortality

**Methods:**

We conducted a prospective cohort study with retrospective analysis at National Taiwan University Hospital system from 2010 to 2024, enrolling hospitalized adults with VRE BSI treated with linezolid or daptomycin (≥ 8 mg/kg). Post hoc minimum inhibitory concentration (MIC) testing for daptomycin was performed by broth microdilution via the Sensititre system which MIC ≥ 8 mg/L indicated resistance.

The primary outcome was 28-day in-hospital mortality; the secondary outcome was recurrent bacteremia defined as same pathogen detected in blood after completed at least 10 days of treatment.

**Figure:**
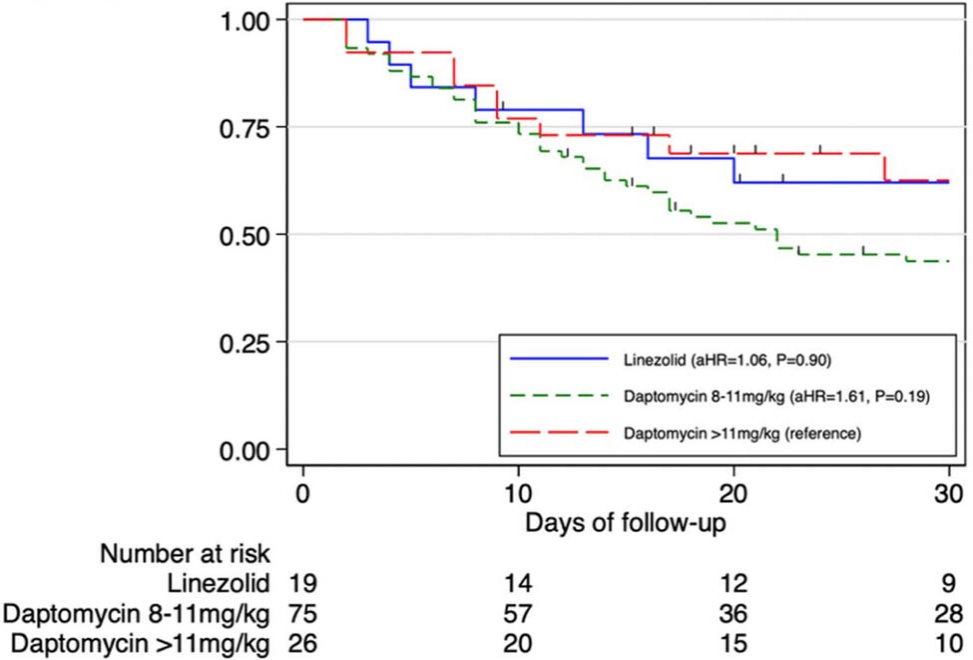
Survival Analysis of 28-day Mortality Categorized by Patients Receiving Linezolid, Daptomycin at 8-11mg/kg Compared to Daptomycin > 11mg/kg

**Results:**

Of 2,230 VRE BSI episodes, 120 isolates met inclusion criteria. Median patient age was 67.3 years (IQR: 55.2–78.4); 57.5% were male. Primary bloodstream infections (45.8%) and urinary tract infections (43.3%) were main sources. Daptomycin treated 101 patients (84.1%), linezolid 19 (15.8%). The 28-day mortality rate was 47.5%. Multivariable analysis showed higher Charlson Comorbidity Index (aOR, 1.30; P=0.02), elevated Pitt bacteremia score (aOR, 1.35; P< 0.01), and lower platelet count (aOR, 0.98; P< 0.01) linked to increased mortality. Compared to daptomycin >11 mg/kg, 8–11 mg/kg doses raised mortality (aOR, 3.30; P=0.04); no difference was seen with high-dose daptomycin vs. linezolid (aOR, 2.02; P=0.39). Recurrent bacteremia occurred in 11 patients, with similar rates in daptomycin and linezolid groups (15.6% vs. 18.2%; P=0.82).

**Conclusion:**

Daptomycin-resistant VRE BSI had high mortality. Higher Charlson Comorbidity Index, Pitt bacteremia score, and lower platelet count predicted mortality. High-dose daptomycin (≥11 mg/kg) may be at least comparable to linezolid in treating daptomycin-resistant VRE BSI, despite in vitro resistance.

**Disclosures:**

All Authors: No reported disclosures

